# Complete mitochondrial genome of the Belligerent sculpin Megalocottus platycephalus (Cottoidei: Cottidae)

**DOI:** 10.1080/23802359.2019.1664348

**Published:** 2019-09-12

**Authors:** Evgeniy S. Balakirev, Alexandra Yu. Kravchenko, Eleonora V. Cherepkova, Pavel A. Saveliev, Alexander A. Semenchenko, Francisco J. Ayala

**Affiliations:** a National Scientific Center of Marine Biology, Far Eastern Branch, Russian Academy of Sciences, Vladivostok, Russia;; b School of Biomedicine, Far Eastern Federal University, Vladivostok, Russia;; c School of Natural Sciences, Far Eastern Federal University, Vladivostok, Russia;; d Russian Academy of Science, Moscow, Russia;; e Far Eastern Federal University, Vladivostok, Russia

**Keywords:** Mitochondrial genome, phylogenetic relationships, Belligerent sculpin *Megalocottus platycephalus*, genetic divergence, Cottidae

## Abstract

The complete mitochondrial (mt) genome was sequenced in two specimens of the Belligerent sculpin *Megalocottus platycephalus* by high-throughput sequencing technology (Ion S5 platform). The sequences are 16,673 bp in size, and the gene arrangement, composition, and size are very similar to the other sculpin mt genomes published previously. Comparison of the two *M. platycephalus* mt genomes now obtained with other complete mt genomes available in GenBank reveals an affinity to the sculpin fishes from the genus *Myoxocephalus*. The intergeneric difference between the *Megalocottus* and *Myoxocephalus* is 0.0757 ± 0.0019, which is significantly less than the corresponding value, 0.1240 ± 0.0120, obtained previously for the sculpin fishes based on the *COI* barcoding marker.

The Belligerent sculpin *Megalocottus platycephalus* (Pallas) has a wide distribution in the North Pacific including the Sea of Japan, the Sea of Okhotsk, the Bering Sea, and the Chukchi Sea (e.g., Fedorov et al. 2003; Mecklenburg et al. 2016). The taxonomical status of *M. platycephalus* is uncertain. It has been considered as a monotypic species, polytypic species including two subspecies, *M. platycephalus platycephalus* and *M. platycephalus taeniopterus*, or as two separate species, *M. platycephalus* and *M. taeniopterus* (review in Radchenko and Petrovskaya 2019). Using the COI, cytochrome b, and 16S rRNA nucleotide sequences, Radchenko and Petrovskaya (2019) revealed the samples of *M. platycephalus* from the different seas form separate groups, with low level of intergroup divergence (*p*-distances <1%). The authors suggested the geographical groups correspond to the southern *M. platycephalus taeniopterus* and northern *M. platycephalus platycephalus* subspecies. To increase the power of phylogenetic analysis of this complex fish group we have sequenced two complete mitochondrial (mt) genomes of *M. platycephalus* (GenBank accession numbers MK936041 and MK936042) from the Sea of Japan, Djigit bay, Kluchi River (27.07.2017, 44.7948°N 136.3413°E, MPL3-17 and MPL5-17). The fish specimens are stored at the museum of the A. V. Zhirmunsky National Scientific Center of Marine Biology, Vladivostok, Russia (www.museumimb.ru) under accession number MIMB 38000.

The genomic DNA was extracted using the KingFisher Flex System and a set of reagents MagMAX DNA Multi-Sample Kit (ThermoFisher Scientific). The complete mt genomes were amplified in five overlapping fragments using the Phusion High-Fidelity DNA Polymerase (ThermoFisher Scientific). Libraries were prepared using Ion Plus Fragment Library Kit and unique adapters (Ion Xpress) with pre-fragmentation on the focused ultrasonicator Covaris M220. Ready libraries were sequenced on the Ion S5 sequencing platform (ThermoFisher Scientific) at the Far Eastern Federal University (Vladivostok, Russia). The complete mt genomes obtained were initially annotated using the MitoFish Web Server (Iwasaki et al. 2013) and further manually adjusted with MEGA 7 (Kumar et al. 2016) by comparisons with mt genomes of other sculpin fishes.

The *M. platycephalus* mt genomes (120× coverage) are 16,673 bp in size; the gene arrangement, composition, and size are very similar to the sculpin fish genomes published previously. There are 47 single nucleotide differences between the two genomes; total sequence divergence (*D*_xy_) is 0.0028 ± 0.0004. Comparison of the two mt genomes now obtained with other complete mt genomes available in GenBank for the genera *Myoxocephalus*, *Enophrys, Icelus*, *Gymnocanthus*, *Mesocottus*, and *Trachidermus* reveals an affinity of *M. platycephalus* to the sculpin fishes of the genus *Myoxocephalus* (Figure 1). The difference (*D*_xy_) between *M. platycephalus* and *Myoxocephalus polyacanthocephalus* + *M. scorpius* is 0.0757 ± 0.0019, which is significantly less than the average intergeneric value, 0.1240 ± 0.0120, obtained previously for sculpin fishes base on the COI barcoding marker (Kartavtsev et al. 2009). Our results indicate that the 5′-COI “barcode” region is not representative for characterizing genetic diversity in sculpin fishes.

**Figure 1.**
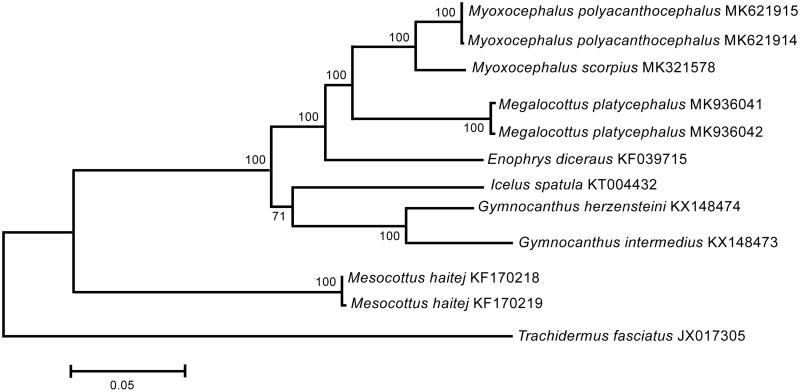
Maximum likelihood tree for the great sculpin *Megalocottus platycephalus* and GenBank representatives of the family Cottidae. The tree is constructed using whole mitochondrial genome sequences. The tree is based on the Hasegawa-Kishino-Yano + gamma + invariant sites (HKY + G + I) model of nucleotide substitution. The numbers at the nodes are bootstrap percent probability values based on 1000 replications.

## References

[CIT0001] Fedorov VV, Chereshnev IA, Nazarkin MV, Shestakov AV, Volobuev VV 2003 Catalog of Marine and Freshwater Fishes from the Northern Sea of Okhotsk. Vladivostok (Russia): Dalnauka; 204 c.

[CIT0002] Iwasaki W, Fukunaga T, Isagozawa R, Yamada K, Maeda Y, Satoh TP, Sado T, Mabuchi K, Takeshima H, Miya M, et al. 2013 MitoFish and MitoAnnotator: A mitochondrial genome database of fish with an accurate and automatic annotation pipeline. Mol Biol Evol. 30:2531–2540. 23955518 10.1093/molbev/mst141 PMC3808866

[CIT0003] Kartavtsev YPh, Sharina SN, Goto T, Balanov AA, Hanzawa N 2009 Sequence diversity at cytochrome oxidase 1 (Co-1) gene among sculpins (Scorpaeniformes, Cottidae) and some other scorpionfish of Russia Far East with phylogenetic and taxonomic insights. Genes Genom. 31:183–197.

[CIT0004] Kumar S, Stecher G, Tamura K 2016 MEGA7: Molecular Evolutionary Genetics Analysis version 7.0 for bigger datasets. Mol Biol Evol. 33:1870–1874. 27004904 10.1093/molbev/msw054 PMC8210823

[CIT0005] Mecklenburg CW, Mecklenburg TA, Sheiko BA, Steinke D 2016 Pacific Arctic Marine Fishes. Conservation of Arctic Flora and Fauna Monitoring Series Report no. 23. Akureyri, Iceland.

[CIT0006] Radchenko OA, Petrovskaya AV 2019 Molecular-genetic differentiation of the Belligerent sculpin *Megalocottus platycephalus* (Pallas, 1814) (Scorpaeniformes: Cottidae). Russ J Mar Biol. 45:56–66.

